# Sodium new houttuyfonate inhibits the growth of *Trichophyton* spp. by potentially inducing oxidative stress and metabolic disruption

**DOI:** 10.3389/fcimb.2026.1880130

**Published:** 2026-08-05

**Authors:** Xiaofang Zheng, Xuemei He, Fangyan Liu, Yonggang Wang, Jingyao Wei, Wenbi Chen, Zhangyong Song

**Affiliations:** 1 School of Basic Medical Sciences, Southwest Medical University, Luzhou, China; 2 Molecular Biotechnology Platform, Public Center of Experimental Technology, Southwest Medical University, Luzhou, China; 3 Hemodynamics and Medical Engineering Combination Key Laboratory of Luzhou, Southwest Medical University, Luzhou, China

**Keywords:** antifungal agents, dermatophytes, energy metabolism, redox balance, sodium new houttuyfonate

## Abstract

**Introduction:**

Dermatophytes are a group of common pathogenic fungi that infect the skin, nails, and hair of humans and other animals, affecting approximately 25% of the global human population. Their antifungal resistance has intensified, underscoring the urgent need for novel antifungal agents. Sodium new houttuyfonate (SNH), a derivative of the *Houttuynia cordata Thunb*., a plant native to Southeast Asian, shows broad-spectrum antimicrobial activity, but its effects on dermatophytes are unclear.

**Methods:**

The *in vitro* activity of SNH against *Trichophyton mentagrophytes* standard strain ATCC MYA-4439 and *Trichophyton rubrum* clinical strain S136 was evaluated through broth dilution assays, growth assays on solid medium, conidia germination rate assessments, biomass quantification, reactive oxygen species (ROS) production assay, with its mechanism further investigated by RNA-seq and qPCR.

**Results:**

SNH exhibited a minimum inhibitory concentration of 100 μg/ml against both strains, significantly suppressing colony expansion, conidia germination, biomass and promoting the production of ROS. Transcriptome analysis revealed SNH upregulated peroxisome biogenesis gene (*PEX5*) while downregulating superoxide dismutase (*SOD*), and dysregulated key energy metabolism genes (*ICL, HK, GLU*).

**Discussion:**

These findings demonstrate that SNH possesses potent antifungal activity against dermatophytes, primarily by disrupting redox homeostasis and interfering with energy metabolism. This provides a theoretical basis for its potential development as an antifungal agent.

## 1 Introduction

Dermatophytes, a group of filamentous and keratinophilic fungi belonging to the family Arthrodermataceae (phylum Ascomycota), infect the skin, nails, and hair of humans and other animals by secreting keratinases that degrade keratin, thereby causing superficial fungal infections known as tinea. In recent years, there has been an increasing number of reported cases of dermatophytosis, with a global prevalence of approximately 25% (Hill et al., 2024). Among the main fungal species involved are *T. rubrum* and *T. mentagrophytes* (Sacheli et al., 2021; Barac et al., 2024). Terbinafine, an inhibitor of squalene epoxidase (SQLE), demonstrates excellent fungicidal efficacy against dermatophytes. Due to its superior safety profile and lower risk of drug interactions compared with fungicidal azoles, terbinafine has been regarded as the “gold standard” in the treatment of dermatophytosis (Stolmeier et al., 2018; Al-Janabi, 2025). However, the prevalence of terbinafine resistance in dermatophytes has been increasing globally, posing a significant public health challenge. In North America, 18.6% of clinical isolates of *Trichophyton* spp. exhibit resistance to terbinafine (Cañete-Gibas et al., 2023; Lockhart et al., 2023). In Europe, 85% of surveyed countries have reported clinical or laboratory-confirmed cases of fungicide-resistant dermatophytosis (Saunte et al., 2021; Hill et al., 2024). On the Indian subcontinent, a newly identified dermatophyte within the *T. mentagrophytes/T. interdigitale* complex, namely *Trichophyton indotineae*, has been recognized as an evolutionarily distinct lineage characterized by high virulence and antifungal resistance. Moreover, it has emerged as a major region for treatment-refractory cutaneous dermatophytosis, accounting for nearly 30% of global terbinafine-resistant cases (Tang et al., 2021; Lockhart et al., 2023; Al-Janabi, 2025). At present, addressing the emergence of antifungal resistance in dermatophytes and promoting the development of novel therapeutic agents remain pressing challenges.

*Houttuynia cordata* Thunb. is widely distributed in Southeast Asia and commonly consumed as food, serving as a typical example of medicinal-edible homology. In traditional Chinese medicine (Zhou et al., 2023), it is believed to clear heat and toxic pathogens from the body. Modern pharmacological analyses have identified its active components as volatile oils, particularly houttuynin and dodecanal. Sodium new houttuyfonate (SNH) is an addition product of dodecanoyl aldehyde from the plant volatile oil and sodium bisulfite, which is more stable. Multiple studies have demonstrated the antimicrobial and anti-inflammatory properties of SNH (Li et al., 2020; Xie et al., 2025; Zhou et al., 2025). Further research has also revealed its antifungal activity against *Candida albicans*, *Candida auris*, *Aspergillus flavus*, and *Aspergillus fumigatus*, along with immunomodulatory effects (Wu et al., 2020; Zhang et al., 2022; Bao et al., 2025; Yang G. et al., 2025). In an *A. fumigatus* infection model, SNH enhanced the phagocytic and fungicidal capacity of alveolar macrophages (Zhou et al., 2025). Despite demonstrated activity against yeasts and molds, no studies have evaluated SNH against dermatophytes. Furthermore, study has shown that SNH augments fungicidal activity by promoting the generation of ROS (Zhou et al., 2025). Accordingly, we hypothesize that the effects of SNH on dermatophytes may be related to this mechanism. These issues are precisely the focus of this study.

This study aims to investigate the effects and underlying mode of action of SNH against dermatophytes. Using assays including microdilution assays, growth experiments on solid medium, conidia germination, and biomass quantification, the *in vitro* antifungal activity of SNH will be evaluated. Furthermore, transcriptome sequencing (RNA-seq) and real-time quantitative PCR (qPCR) will be employed to elucidate the potential mechanisms of action. The objective is to determine whether SNH exhibits inhibitory effects on dermatophytes and, if so, to explore its mechanistic basis.

## 2 Materials and methods

### 2.1 Strains and cultures

The experimental strains used in this study were a clinical isolate of *T. rubrum* designated as S136 (hereafter abbreviated to S136) and the standard strain *T. mentagrophytes* ATCC MYA-4439 (hereafter abbreviated to TM). The S136 strain was obtained from the collection of the Pathogenic Fungal Infection and Immunology Research Group at Southwest Medical University, Luzhou, Sichuan Province, China, while the *T. mentagrophytes* strain was kindly provided by Professor WuDian Xiao from Southwest Medical University (Wang et al., 2022). The strains were cultured on Potato Dextrose Agar (PDA) medium at 28°C for 7–14 days. Conidia were collected by filtration through a 50-µm mesh sieve and suspended in sterile water containing 0.05% (v/v) Tween-80. The concentration was adjusted to 10^6^ conidia/ml using a hemocytometer and this suspension was used as the stock solution.

### 2.2 Drugs and reagents

Sodium new houttuyfonate (SNH, purity ≥ 98%, catalog No. B13024) was purchased from BIOBOMEI (Wuhan, China). Voriconazole (VOR, CAS No. 137234-62-9, purity ≥ 99%) was obtained from Macklin (Shanghai, China). SNH was dissolved in sterile distilled water to prepare a stock solution of 10 mg/mL, while VOR was dissolved in dimethyl sulfoxide (DMSO) to a stock solution of 5.12 mg/ml. Both stock solutions were stored at -20 °C and freshly diluted in culture medium prior to each experiment.

### 2.3 Minimum inhibitory concentration assay

The experimental procedure was conducted in accordance with the Clinical and Laboratory Standards Institute document (Institute CaLS, 2017). VOR, used as a positive control azole drug, was tested at concentrations ranging from 0.0625 to 2 µg/ml (Deng et al., 2015; Institute CaLS, 2017), while SNH was tested at concentrations ranging from 25 to 800 µg/ml. Drugs were subjected to a twofold serial dilution in 96-well plates. Each well received 100 µL of the drug solution and 100 µL of the conidial suspension. After static incubation at 35 °C for 4 days, the plates were visually inspected and the optical density at 600 nm (OD600) was measured. The MIC values (the concentration required to achieve 80% inhibition of the growth of the test organism) were subsequently calculated. The equation used was as follows:


$$\text{inhibition rate }\left(\%\right)=\left[\left(\text{O}{\text{D}}_{\text{control}}-\text{O}{\text{D}}_{\text{experimental}}\right)/\left(\text{O}{\text{D}}_{\text{control}}-\text{O}{\text{D}}_{\text{blank}}\right)\right]\times 100$$

OD _control_ represents the optical density measured in the control wells containing only the conidial suspension, while OD _experimental_ refers to the optical density measured in the test wells containing both the conidial suspension and the corresponding drug concentration. OD _blank_ denotes the optical density measured in the blank (negative) control wells containing only the culture medium.

### 2.4 Effect of SNH on colony growth on solid medium plates

The experimental protocol was adapted with minor modifications from a previously described method (Shen et al., 2024). Briefly, fungal plugs (8 mm in diameter) were obtained from 14-d-old mature cultures on PDA using a sterile punch. These plugs were placed with the mycelial surface facing upward onto solid PDA plates of the respective composition. The control group plates contained 1 µg/ml VOR. The SNH test group plates contained concentrations of 50, 100, or 200 µg/ml SNH, corresponding to final concentration gradients of 1/2 × MIC, MIC, and 2 × MIC of SNH, respectively. A negative control group consisted of solid PDA plates without any supplements. The growth status was monitored and photographed every three days, while the colony diameter was measured daily using a Vernier caliper.

### 2.5 Effect of SNH on conidia germination rate of dermatophytes

The conidia germination rate assay was performed using a previously described method, with minor modifications (Zeng et al., 2015). Briefly, SNH was dissolved in water and subsequently mixed with Roswell Park Memorial Institute (RPMI) medium to prepare liquid culture media containing 50, 100, or 200 µg/ml SNH. The positive control, negative control, and test groups corresponded to those described in growth experiments on solid medium. The final working concentration of conidial suspensions was 5×10^5^ conidia/ml. The tubes were inverted 3–4 times to ensure thorough mixing and subsequently incubated at 28°C for 24 h. Microscopic examination was performed to count 200 conidia at random and determine whether each conidia had germinated, so that the germination rate could be calculated.

### 2.6 Effect of SNH on the biomass of dermatophytes

The positive control, negative control and test groups corresponded to those described in subsection 3. Conidial suspensions of TM or S136 were respectively inoculated into 25 ml of liquid potato dextrose broth medium containing the corresponding drug concentrations, yielding a final conidial concentration of 5×10^5^ conidia/ml. The liquid cultures were incubated at 28°C with shaking (200 rpm). After 7 d incubation, the liquid medium containing mycelia was filtered through sterile filter paper. The harvested mycelia were then centrifuged (12000 rpm, 20 minutes) to remove the supernatant, transferred into pre-weighed centrifuge tubes, oven-dried at 60°C for 10 h, and weighed again.

Biomass = W1−W2.

W1: weight (g) of dried centrifuge tube (with sample); W2: weight (g) of corresponding empty centrifuge tube.

### 2.7 Transcriptome sequencing

Conidial suspensions of strains TM or S136 were respectively prepared in liquid PDA medium without any drug treatment, achieving a final concentration of 10^6^ conidia/ml. The culture was incubated at 28°C with shaking (200 rpm) for 5 d. Subsequently, SNH was added to achieve a subinhibitory concentration equivalent to 70% of the MIC (Lopes et al., 2022). After thorough mixing, the culture was incubated with shaking for a further 1 h or 6 h. The mycelium was then collected by filtration and centrifugation, snap-frozen in liquid nitrogen, and transported on dry ice to Qingdao Biomarker Technologies Co., Ltd. (Qingdao, China; https://www.biocloud.net) for transcriptome sequencing. RNA concentration and purity were measured using a NanoDrop 2000 spectrophotometer (Thermo Fisher Scientific, Wilmington, DE, USA). RNA integrity was assessed using the RNA Nano 6000 Assay Kit of the Agilent Bioanalyzer 2100 system (Agilent Technologies, Santa Clara, CA, USA). The libraries were sequenced on an Illumina NovaSeq platform (Illumina, San Diego, CA, USA) to generate 150-bp paired-end reads. The experiment included three biological replicates, each consisting of independently cultured mycelial samples. Each sample was subjected to independent library construction for sequencing. The raw reads were further processed using the bioinformatic pipeline tool, the BMKCloud online platform. Genes with fold change (FC) ≥ 2 and false discovery rate (FDR)< 0.01 were considered to be significantly differentially expressed. Raw sequence data were deposited in the National Center of Biotechnology Information (accession number: PRJNA1380017).

### 2.8 qPCR analysis

Samples were collected using the same method as for transcriptome sequencing, with repeated grinding in liquid nitrogen. RNAiso Plus (Takara Bio, Shiga, Japan) was added to extract total RNA. RNA concentration was measured, and reverse transcription was performed for each sample, using the PrimeScript™ FAST RT Reagent Kit with gDNA Eraser (Takara Bio). For each sample, 1 μg of RNA was reverse-transcribed into cDNA. The primer sequences used for different genes are listed in **Table 1**. The *β-tubulin* gene of strains TM and S136 were used as the respective internal reference gene (Petrucelli et al., 2023; Yang SQ. et al., 2025). Relative expression was quantified using the 2^−ΔΔCT^ method (Vandesompele et al., 2002).

**Table 1:** Primer sequences for genes.

Strain	Gene	Forward primer	Reverse primer
TM	*β-tubulin*	ACATGGTTCCATTCCCTCGT	ATCGAACATCTGCTGGGTCA
TM	*PEX1*	GTCGCTTTGTCTTCGCTCTT	TCTTCACTGCTGGCTTCTGA
TM	*PEX2*	CACTGGCGATGAGAACAAGG	ATAACGTCGCTCTCCGAAGT
TM	*PEX3*	GAGGCTGCAGGATCAGAGAT	ACGACCGCGTTAAAGAGTTG
TM	*PEX14*	CATTGAAGGAGCCCGTGAAG	CTGGTCGAGTAGTGGCAGAT
TM	*SOD*	GACAGCACCACTACCACAAC	AATGGTAACTGCCTGACCGA
TM	*ICL*	ATCGTGGCTCCCAACTTGTA	GTGGCAATCGGGTTAGCAAT
TM	*hexokinase*	TAGCTACCTCGCGATCAGAC	GGAGTCCAGAGCCATGAGTT
TM	*β-glucosidase*	ACCTATGGCCCTTTGCTGAT	GTTCGCCCTTGAGTAGCTTG
TM	TMEN_3035	CTTCATGGGCGCAGAATTGA	CAGCACCCATGGATACAAGC
TM	TMEN_987	GACACCTATCCTCACGCA	TCCACATGGCAAGGTCGTAT
TM	TMEN_4188	GTATGACTTTGGCGGACCAC	GCACCCAAAGGCAAAGAAGA
S136	H107_01813	ACAGTGAGAAGAAGGCCGAA	GTCGTAGTAGGTCGCTCCAA
S136	H107_03526	CTCACGCAAATTCGGGACAT	CGTAAGTCCACATGGCAAGG
S136	H107_08034	CGTCCCTTCTAAGCCCATCT	TTCCACACCCCAGAACTACT
S136	*β-tubulin*	GCCATACAACGCCACTCTTT	GGGTTAGTGAGCTTGAGGGT
S136	*PEX5*	ATCCATGTGAGCCAGAACCA	ACGACCCATCTGGCTAAACA
S136	*PEX7*	AACCAACCACCTCACATTGC	CAGTGGCAGGTGTAAATCCG
S136	*PEX14*	CAGGATCCCTCTGTCTCGTC	TCTCTTCCTGCGTCAGGTTT
S136	*PEX19*	GAGTGACTTTGGGACTTCGC	CCGTCTTGACAAGCTGATGG
S136	*SOD*	AGAACTCTGGTGGTGGTGAG	GGATACCAGCCAAAGCAGTG
S136	*ICL*	ATGTTTCTGGTTGGCAGAGC	TGGAACAGTTGAGCCAGGAA
S136	*hexokinase*	ACTCTCTCTGGCGTCTAAGC	CCGGGTACTTGTTGATGACG
S136	*β-glucosidase*	CGTACTGGATACGAGCGAGA	TTGAGCGATGCAGTTTAGCC

### 2.9 ROS production assay

A pre-prepared spore suspension was added to a 96-well plate. SNH solution was then added to reach the target concentrations, and the corresponding volume of culture medium was added to the blank group. The final volume in each well was 200 μl. After vortex mixing, the plate was incubated statically at 28°C for 1 or 6 hours. The experimental groups included a blank control group and SNH treatment groups at 50, 100, or 200 µg/ml. After incubation, the ROS fluorescent probe DCFH-DA (working concentration of 10 μmol) was added, and the plate was incubated in the dark for 30 minutes. Fluorescence intensity was then measured at excitation/emission wavelengths of 488/525 nm.

### 2.10 Statistical analysis

Data analysis was performed using GraphPad Prism software (version 9.5) (GraphPad Software, San Diego, CA, USA). Group comparisons were conducted using one-way analysis of variance (ANOVA) (for comparisons of more than two samples). Each experiment was performed with three biological replicates, and *p<* 0.05 was considered to be statistically significant.

## 3 Results

### 3.1 The antifungal activity of SNH against strains TM and S136

**Figure 1:**
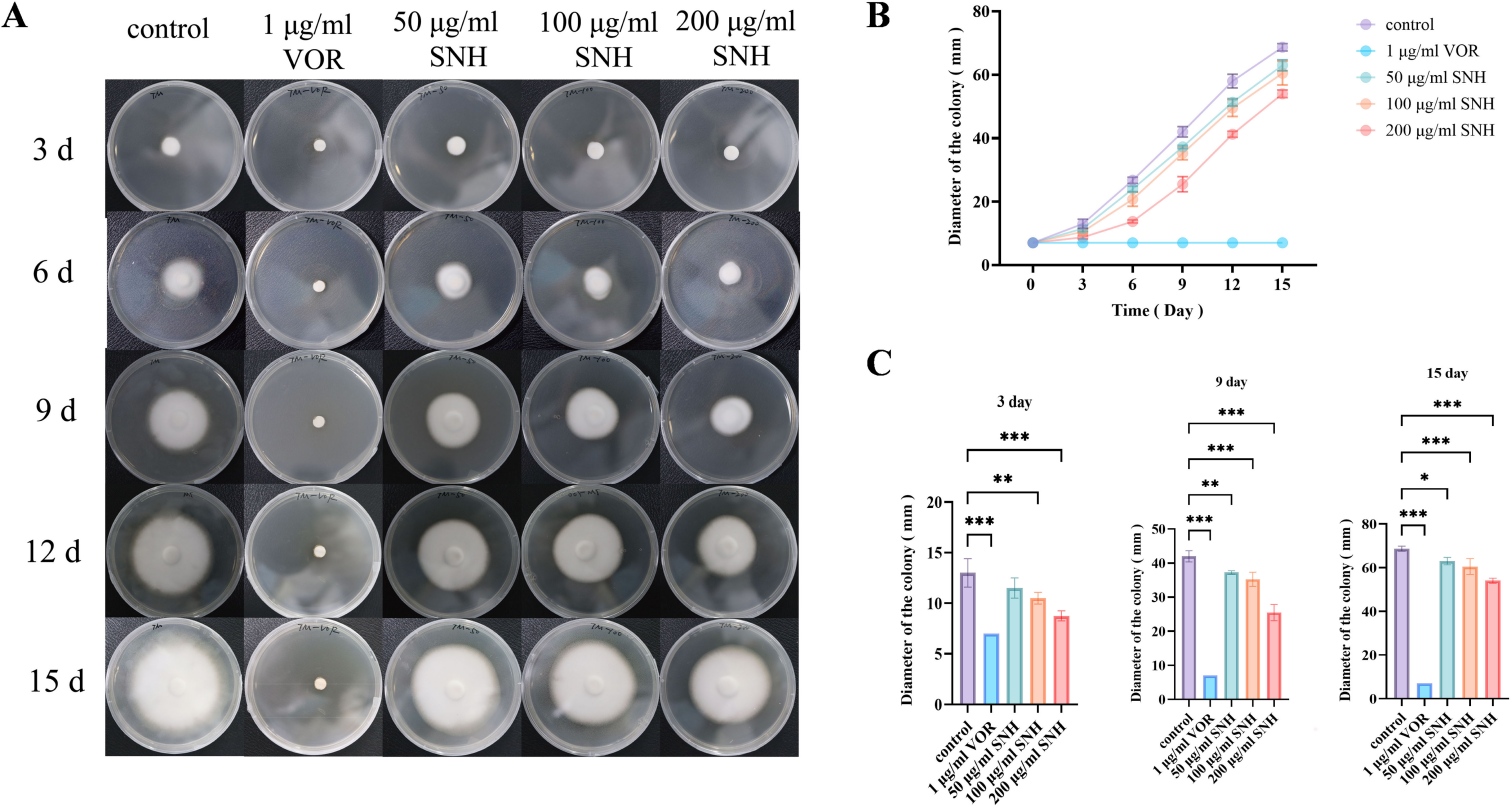
Inhibitory effect of SNH on the growth of *T. mentagrophytes* (TM). **(A)** Colony growth of strain TM on plates of solid medium under various drug concentrations over a 15-day period. **(B)** Temporal changes in colony diameter (cm). **(C)** Statistical analysis of colony diameter differences on days 3, 9, and 15. Negative control: an 8-mm diameter fungal plug was incubated on drug-free solid PDA medium at 28°C and growth was monitored for 15 d. Positive control: an 8-mm diameter fungal plug was cultured on solid PDA plates containing 1 μg/ml VOR for 15 d. Experimental groups: an 8-mm diameter plug was incubated on solid PDA supplemented with 50, 100, or 200 μg/ml SNH and cultured for 15 d. Data analysis was conducted using one-way analysis of variance (ANOVA). Each experiment was performed with three biological replicates. Significance levels: ns, *p* > 0.05; *p< 0.05; **p< 0.01; ***p< 0.001. PDA, potato dextrose agar; VOR, voriconazole; SNH, sodium new houttuyfonate.

Results from the liquid culture microdilution assay indicated that the MIC of VOR for strain TM was< 0.25 μg/ml, while that for strain S136 was< 0.125 μg/ml. The MIC of SNH against both strain TM and S136 was 100 μg/ml. Based on these results, 100 μg/ml was defined as the MIC for both strains; accordingly, 50 μg/ml and 200 μg/ml were selected as the 1/2 × MIC and 2 × MIC of SNH for both strains. To further test the antifungal activity of SNH, fungal plugs (8 mm in diameter) were inoculated onto solid PDA plates containing various concentrations of SNH and fungal growth was monitored for growth. In the negative control group of the TM strain, mycelial growth around the plugs was visible by day 3, appearing as fluffy colonies (**Figure 1A**). With prolonged incubation, the colony diameter increased progressively (**Figure 1B**). By day 15, the colonies had covered more than two-thirds of the plate surface area, with peripheral hyphal extension observed. In contrast, SNH-treated groups showed markedly inhibited fungal growth at the same time points (**Figure 1A**). The colonies appeared more compact in the presence of SNH, with increased transparency toward the plate edges and no significant hyphal extension (**Figure 1A**). Statistical analysis of colony diameters across groups revealed that, at days 3, 9, and 15, all colonies of SNH-treated groups were significantly smaller colonies than the colonies on the negative control plates (**Figure 1C**).

**Figure 2:**
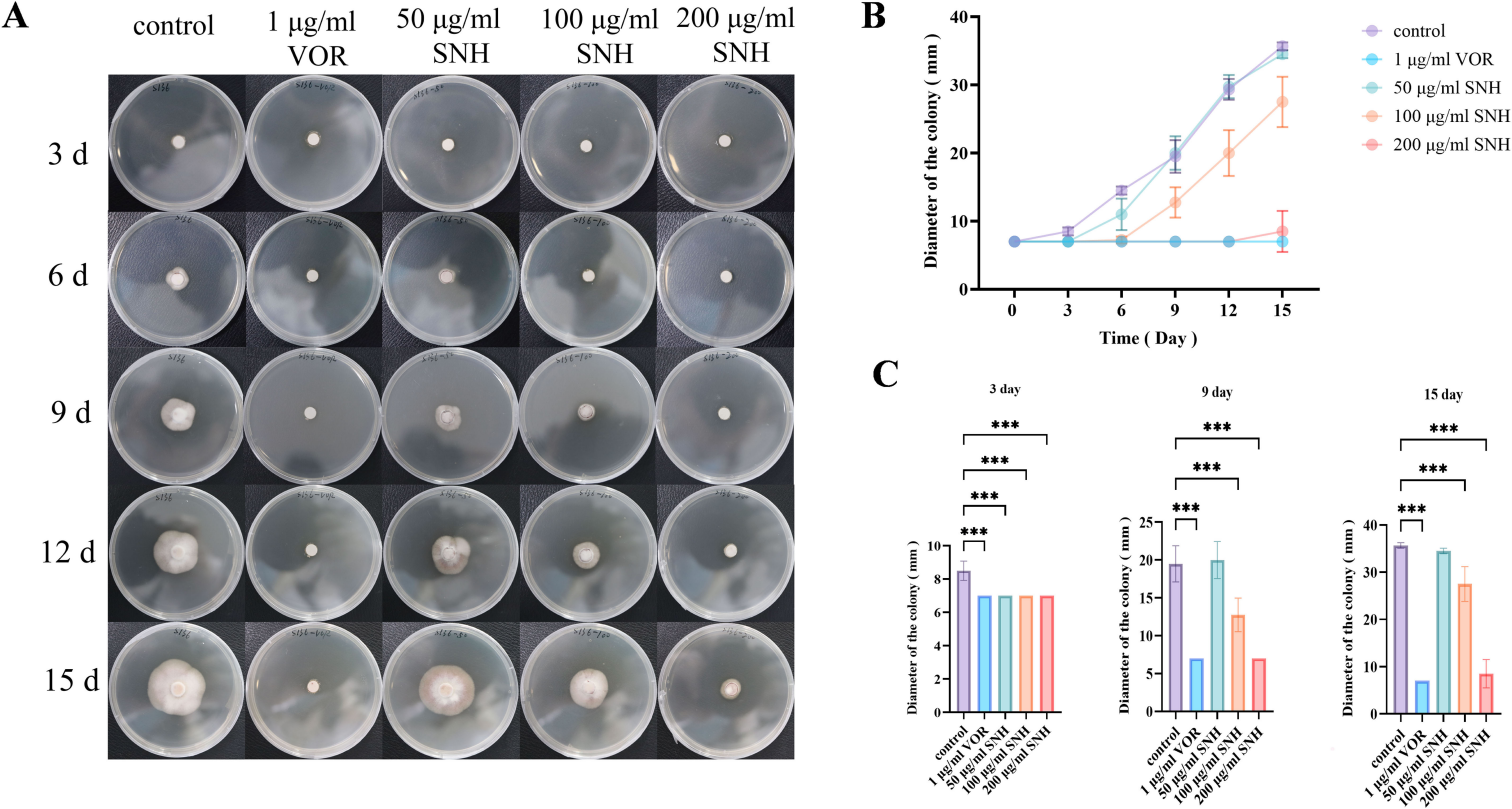
Inhibitory effect of SNH on the growth of *T. rubrum* S136. **(A)** Colony growth of strain S136 on plates of solid medium under various drug concentrations over a 15-day period. **(B)** Temporal changes in colony diameter (cm). **(C)** Statistical analysis of colony diameter differences on days 3, 9, and 15. Negative control: an 8-mm diameter fungal plug was incubated on drug-free solid PDA medium at 28°C and growth was monitored for 15 d. Positive control: an 8-mm diameter fungal plug was cultured on solid PDA plates containing 1 μg/ml VOR for 15 d. Experimental groups: an 8-mm diameter plug was incubated on solid PDA supplemented with 50, 100, or 200 μg/ml SNH and cultured for 15 d. Data analysis was conducted using ANOVA. Each experiment was performed with three biological replicates. Significance levels: ***p< 0.001. PDA, potato dextrose agar; VOR, voriconazole; SNH, sodium new houttuyfonate.

For strain S136, distinct colony formation was observed by 6 d culture in the untreated control group (**Figure 2A**), with the colony diameter increasing to 35 mm by day 15. Compared with the negative control, all SNH-treated groups exhibited marked suppression of colony growth (**Figure 2B**). In addition, no colony growth was detected in the VOR-treated positive control group throughout the entire incubation period. Statistical analysis of colony diameters at various time points (days 3, 9, and 15) confirmed that all SNH concentrations significantly inhibited the growth of strain S136 (**Figure 2C**). Further analysis revealed that there was a progressive delay in the onset of visible colony growth with increasing SNH concentrations; growth was observed on day 6 in the negative control group, day 9 in the 1/2 × MIC group, day 12 in the MIC group, and only minimal growth around the original fungal plug by day 15 in the 2 × MIC group. In addition, based on the results of the solid spot assay and the colony diameter measurements, the inhibitory effect of SNH on strain S136 appeared to be stronger than that on strain TM.

### 3.2 SNH can inhibit the germination rate and biomass production of strains TM and S136

**Figure 3:**
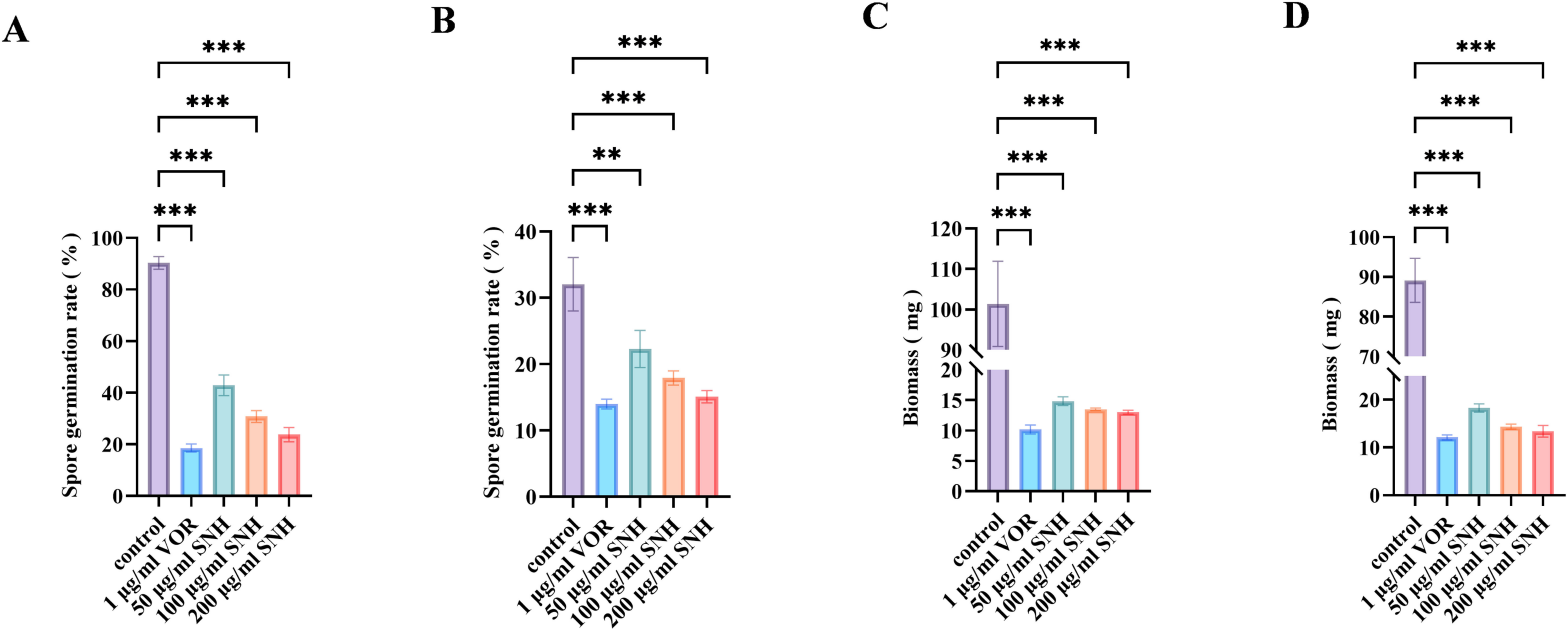
Inhibitory effect of SNH on the conidia germination rate of strains *T. mentagrophytes* TM and *T. rubrum* S136. **(A, B)** Conidia germination rates of strains TM **(A)** and S136 **(B)** after 24 h of liquid shaking culture at 28°C in the presence of different SNH concentrations. **(C, D)** Biomass dry weight (g) of TM **(C)** and S136 **(D)**, after 7 days of liquid shaking culture at 28°C in the presence of different SNH concentrations. Data analysis was conducted using ANOVA. Each experiment was performed with three biological replicates. Significance levels: **p< 0.01; ***p< 0.001.

To further investigate how SNH affects the growth of dermatophytes, liquid media containing different drug concentrations were prepared to culture the fungal strains at 28°C with shaking for 24 h, after which the proportion of germinated conidia was counted under a microscope. In the untreated negative control group, the conidia germination rate of strain TM reached over 80% at 24 h. In contrast, under treatment with 1/2 × MIC of SNH, the germination rate fell to 42%, and it progressively decreased with increasing SNH concentrations, reaching only 23% at 2 × MIC (**Figure 3A**). Similarly, with strain S136, the germination rate in the untreated negative control group exceeded 30%, but treatment with SNH at the MIC concentration resulted in a germination rate of only 17.9%, which further declined to 15.1% at 2 × MIC (**Figure 3B**). Based on these findings, we measured the biomass after 7 days of shaking incubation at 28°C The results showed that the mean ± standard deviation (SD) dry biomass of strain TM in the untreated control exceeded 101.4 ± 10.51 mg, while SNH treatment significantly reduced biomass accumulation. At 1/2 × MIC, the biomass was only 14.83 ± 0.709 mg (**Figure 3C**). Similarly, the biomass of strain S136 fell significantly from 89.13 ± 5.55 mg in the untreated negative control group to below 18.276 ± 0.862 mg under 1/2 × MIC of SNH treatment (**Figure 3D**). These data showed that the significant suppression of dermatophyte growth by SNH involved inhibition of conidia germination rate and reduced biomass production.

### 3.3 The mechanism of the antifungal effect of SNH on the TM strain

**Figure 4:**
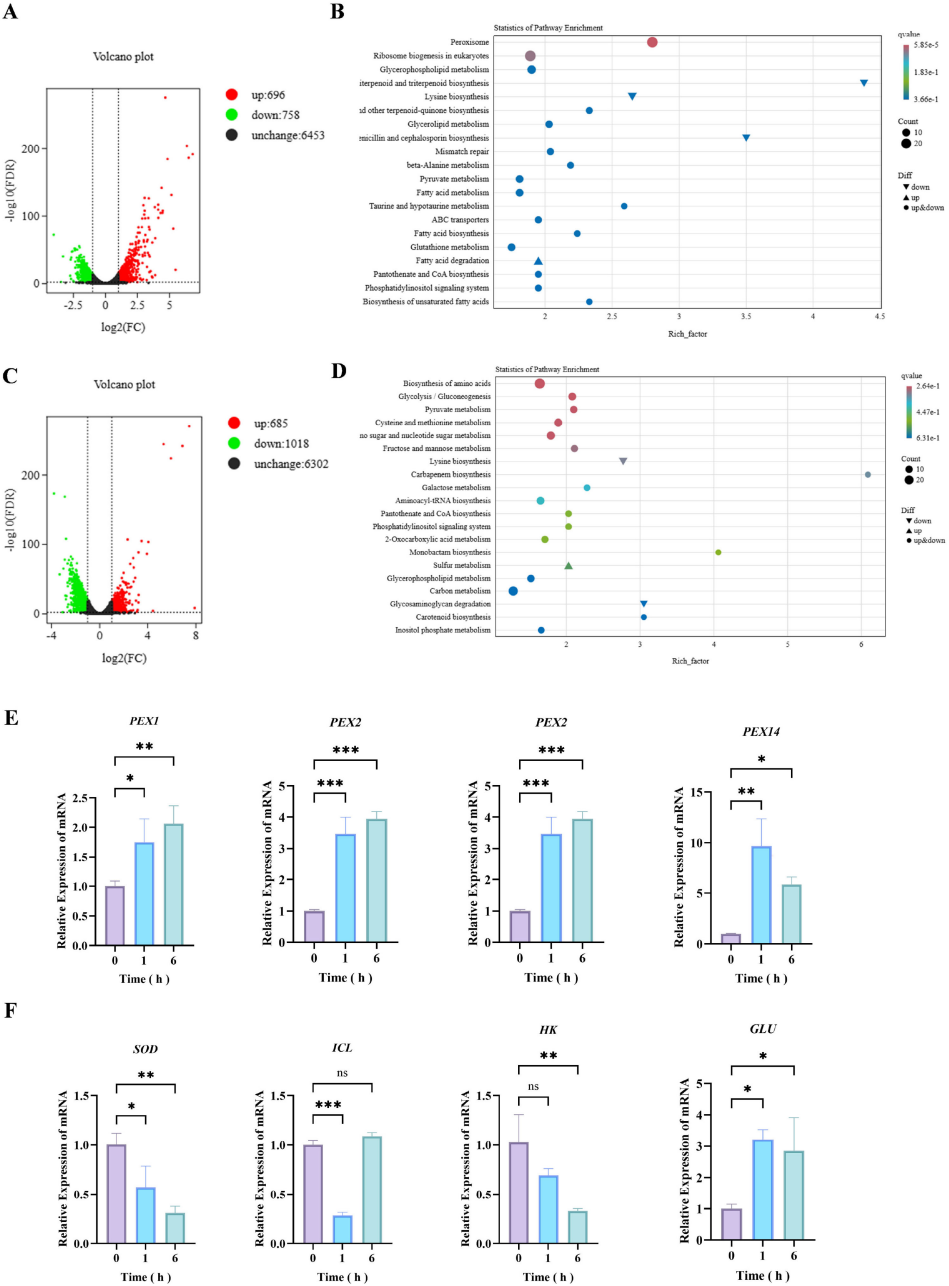
Transcriptome sequencing and real-time quantitative PCR (qPCR) analysis of the effect of SNH on *T. mentagrophytes* strain TM. **(A, C)** Volcano plots of differentially expressed genes (DEGs) identified by RNA-seq in TM treated with a subinhibitory concentration of SNH (70% MIC, 70 μg/ml) for 1 h **(A)** or 6 h **(C)**; FC, fold change; FDR, false detection rate. **(B, D)** Kyoto Encyclopedia of Genes and Genomes (KEGG) enrichment analysis of RNA-seq data from strain TM exposed to SNH for 1 h **(B)** or 6 h **(D)**. **(E, F)** qPCR analysis of DEGs in strain TM at different time points. Data analysis was conducted using ANOVA. Each experiment was performed with three biological replicates. Significance levels: ns, *p* > 0.05; *p< 0.05; **p< 0.01; ***p< 0.001.

To further clarify the antifungal mechanism of SNH, mature hyphae of dermatophytes were exposed to a sub-inhibitory concentration (70% MIC, 70 μg/ml) of SNH for 1 h or 6 h before transcriptome sequencing was performed. After 1 h of SNH treatment, TM exhibited 696 up-regulated genes and 758 down-regulated genes, relative to the no-SNH negative control (**Figure 4A**). Kyoto Encyclopedia of Genes and Genomes (KEGG) enrichment analysis at this stage showed that the differentially expressed genes (DEGs) were mainly concentrated in peroxisome pathways (**Figure 4B**; **Supplementary Figure 1**). This suggested that SNH rapidly (within 1 h) interfered with the oxidative homeostasis balance of the cell at an early stage. After the treatment time was extended to 6 hours, the changes in gene expression intensified, with a total of 685 up-regulated genes and 1018 down-regulated genes in SNH-treated TM (**Figure 4C**). Pathway enrichment analysis showed (**Figure 4D**; **Supplementary Figure 2**) that the focus of pathway enrichment following extended exposure to SNH shifted to core metabolic pathways, such as carbon metabolism and biosynthesis of amino acids (**Figure 4D**), indicating that sustained treatment with SNH led to a profound disruption of the basic energy metabolism and biosynthetic processes in strain TM. However, qPCR results showed no significant differences in ergosterol biosynthesis-related genes at both 1 hour and 6 hours (**Supplementary Figure 3**).

To test the above findings, quantitative analysis on the expression of representative genes in the key pathways was conducted (**Figure 4E**; **Supplementary Table 1**). In terms of oxidative stress response, peroxisome-related genes (*PEX1, PEX2, PEX3, PEX14*) were upregulated at both 1 h and 6 h; *PEX* genes encode peroxins, which are proteins essential for the biogenesis and function of peroxisomes, and peroxisomes participate in intracellular ROS detoxification). Expression of the key enzyme gene responsible for scavenging superoxide radicals, superoxide dismutase gene (*SOD*), was significantly decreased over time, indicating that SNH inhibited the “detoxification” response of the TM strain to external stress stimuli (**Figure 4E**). In addition, the expression levels of *isocitrate lyase (ICL)*, *hexokinase (HK)*, and *β-glucosidase (GLU)*, which are involved in other energy metabolism pathways, changed to varying degrees over time, confirming the functional disorder of the energy metabolism network in TM caused by SNH antifungal activity (**Figure 4F**).

### 3.4 The mechanism of the antifungal effect of SNH on the S136 strain

**Figure 5:**
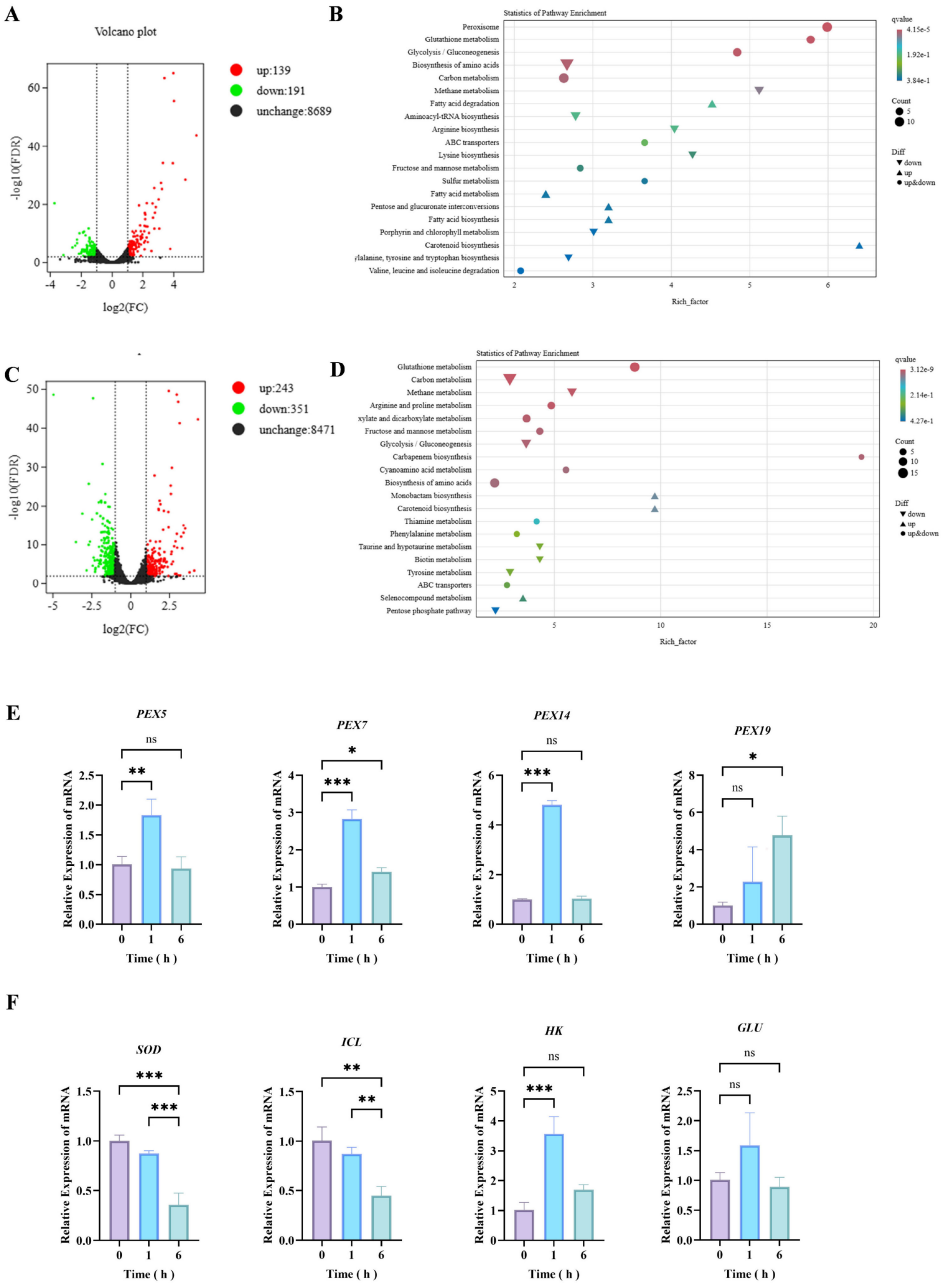
Transcriptome sequencing and qPCR analysis of the inhibitory effect of SNH on *T. rubrum* strain S136. **(A, C)** Volcano plots of DEGs identified by RNA-seq in strain S136 treated with a subinhibitory concentration of SNH (70% MIC, 70 μg/ml) for 1 h **(A)** or 6 h **(C)**; FC, fold change; FDR, false detection rate. **(B, D)** KEGG enrichment analysis of RNA-seq data from strain S136 exposed to SNH for 1 h **(B)** or 6 h **(D)**. **(E, F)** Quantitative expression (qPCR) analysis of DEGs in S136 at different time points. Data analysis was conducted using ANOVA. Each experiment was performed with three biological replicates. Significance levels: ns, *p* > 0.05; *p< 0.05; **p< 0.01; ***p< 0.001.

The volcano plot revealed that treatment with SNH for 1 h resulted in 139 up-regulated and 191 down-regulated genes, compared with the no-SNH negative control (**Figure 5A**). KEGG pathway enrichment analysis of the DEGs indicated that several metabolic and biosynthetic pathways were significantly enriched, including peroxisomes, carbon metabolism, biosynthesis of amino acids, and glutathione metabolism, suggesting that SNH broadly influences stress response mechanisms and core metabolic processes during the initial (1 h) phase of treatment (**Figure 5B**; **Supplementary Figure 1**). However, the enrichment of these related pathways did not map to the ergosterol biosynthesis pathway, and the qPCR results also showed no significant differences (**Supplementary Figure 3**). After 6 h of SNH exposure, the number of DEGs further increased to 243 up-regulated and 351 down-regulated genes (**Figure 5C**). Enrichment analysis at this stage showed that SNH primarily impacted key metabolic pathways such as carbon metabolism, glutathione metabolism, and biosynthesis of amino acids (**Figure 5D**; **Supplementary Figure 2**). The marked down-regulation of genes involved in central carbon metabolism and energy metabolism indicated that prolonged SNH treatment progressively impaired the basic energy production and biosynthetic capacity of the S136 strain, potentially leading to cellular oxidative stress damage and growth abnormalities.

To validate these observations from transcriptome sequencing, the expression levels of oxidative stress-related genes in the S136 strain were quantified. The expression of genes *PEX5*, *PEX7*, and *PEX14* (**Figure 5E**; **Supplementary Table 1**) increased significantly after 1 h exposure to SNH and decreased thereafter, whereas *PEX19* expression remained higher than that in the absence of SNH at both 1 h and 6 h. The expression of the *SOD* gene declined substantially over time. This expression pattern suggests that, although the S136 strain attempted to induce certain stress responses when exposed to SNH, its core antioxidant detoxification capacity was ultimately suppressed by SNH (**Figure 5E**). In energy metabolism, key carbon metabolic enzyme genes *ICL*, *HK*, and *GLU* also exhibited significant expression alterations (**Figure 5F**), confirming that SNH disrupts the energy metabolism network in the S136 strain.

**Figure 6:**
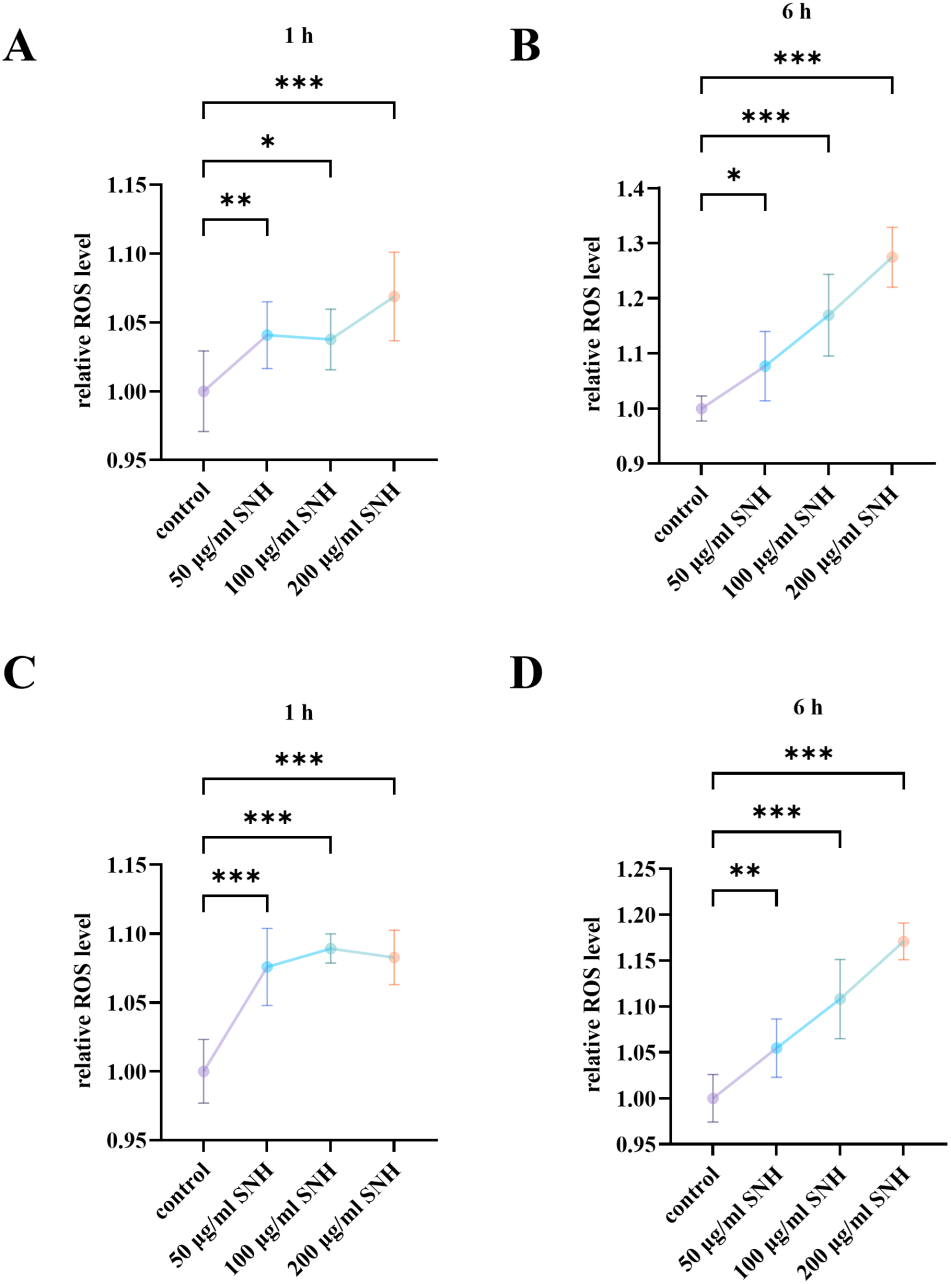
ROS levels under SNH treatment at different time points. ROS level in *T. mentagrophytes* was measured at 1 hour **(A)** and 6 hours **(B)** of SNH treatment. ROS level in *T. rubrum* was measured at 1 hour **(C)** and 6 hours **(D)** of SNH treatment. Data analysis was conducted using ANOVA. Significance levels: ns, *p* > 0.05; *p< 0.05; **p< 0.01; ***p< 0.001. SNH, sodium new houttuyfonate; ROS, reactive oxygen species.

Based on the observed changes in peroxisome-related genes and antioxidant genes in both strains, this study further measured intracellular ROS levels under SNH treatment. The results (**Figure 6**) showed that at 1 hour of SNH treatment, ROS levels in dermatophytes increased, with some variation among different SNH concentrations. At 6 hours, SNH significantly increased intracellular ROS production. These findings indicate that SNH markedly disrupts the redox homeostasis of dermatophytes, suggesting that disturbing intracellular redox balance is one of the key mechanisms by which SNH exerts its anti-dermatophyte activity.

## 4 Discussion

Over recent years, the global incidence of dermatophytosis has been rising, currently affecting approximately 25% of the world’s population (Hill et al., 2024). The infection can manifest in various regions, including the skin (tinea corporis), groin (tinea cruris), and scalp (tinea capitis), with clinical presentations ranging from asymptomatic to acute inflammation or chronic refractory infections (Gnat et al., 2021; Su et al., 2022). Current treatment primarily relies on antifungal agents such as allylamines (terbinafine) and azoles (itraconazole). However, the incidence of resistance to antifungals in dermatophytes is increasing worldwide, driven largely by mutations in the target genes of these antifungals, such as *SQLE* and *CYP51A (*Shao et al., 2025). Together with the challenge of difficult-to-treat infections, this trend has elevated dermatophytosis to a serious public health concern. Therefore, the development of novel therapeutic agents, ideally with multiple target sites in the fungus to slow or prevent the evolution of resistance, represents a research area of considerable importance. The present study investigates the antifungal effects and underlying mechanisms of SNH against dermatophytes, with the aim of providing a theoretical foundation for the development of new antifungal drugs. Among the causal agents of dermatophyte infections, *T. rubrum* is the most common species, representing up to 60.3% of cases (Sacheli et al., 2021), whereas the *T. mentagrophytes* complex accounts for about 11.2% of cases and is frequently associated with antifungal resistance due to its high mutation rate (Gupta et al., 2023). To thoroughly assess the potential of SNH, we selected *T. rubrum* strain S136 and *T. mentagrophytes* ATCC MYA-4439 (strain TM) as study subjects to ensure the broad applicability of our findings. However, it should be noted that the use of a single isolate for each species represents a limitation of this study. The observed phenotypes and mechanisms may not fully capture the intraspecies variability, and crucially, the activity of SNH against clinical isolates with existing terbinafine resistance remains to be evaluated. Therefore, to systematically evaluate the activity of SNH against clinical dermatophyte isolates from diverse geographical origins and with various resistance profiles (such as terbinafine resistance), further investigations on more clinical isolates are essential in future. Our results demonstrated that SNH exhibits significant inhibitory activity against both strains, with a consistent MIC of 100 µg/ml. This value differs from previously reported MICs of SNH for *C. auris* (32–64 µg/ml) (Yang G. et al., 2025), *Cryptococcus neoformans* (32 µg/ml) (data not published), and *A. fumigatus* (50–100 µg/ml) (Zhang et al., 2022). Based on the solid and liquid investigations demonstrate that SNH exerts its effect as a fungistatic activity. It is worth noting that the inhibitory effect of SNH on the TM and S136 strains was not entirely consistent (**Figures 1**, **2**): the clinical strain S136 appeared to reduce growth and conidia germination in response to SNH more than did in the TM. Additionally, in *A. fumigatus*, SNH inhibits the fungus by suppressing ergosterol synthesis and affecting the expression of related genes (Zhang et al., 2022), whereas in the dermatophytes examined here, transcriptomic enrichment analysis and qPCR analysis of these genes involved in ergosterol biosynthesis (Yu et al., 2007; Bhattacharyya et al., 2023) revealed no significant changes (**Supplementary Table 2**; **Supplementary Figure 3**). These differences highlight species-specific variations in sensitivity to SNH, underscoring the need for further research into the mechanism of action of SNH across different fungal pathogens.

Research on *T. rubrum* has demonstrated that biofilm formation leads to thickening of the extracellular matrix, thereby restricting drug diffusion (Costa-Orlandi et al., 2021; Samdavid Thanapaul et al., 2023), and this dense hyphal aggregation constitutes a physical barrier developed by the fungus to withstand SNH stress. Beyond fostering antifungal resistance, the hyphal phase is central to the pathogenicity of dermatophytes and plays a key role in triggering host immune and inflammatory responses (Deng et al., 2023). In the current study, SNH inhibited hyphal extension at the colony periphery on solid media, which not only restricts fungal growth but is also likely to impair the ability of the fungi to invade host tissues and establish effective infections (**Figures 1**, **2**). Our findings are consistent with earlier reports on SNH effects in *A. fumigatus*, which showed that SNH significantly inhibited conidia germination, hyphal growth, and biofilm formation (Zhang et al., 2022). This similarity suggests that the antifungal mechanism of SNH may be conserved across different fungal genera, primarily by blocking the critical transition from conidia to invasive hyphae. Future studies should focus on identifying the specific molecular targets of SNH and evaluating the efficacy of SNH in more complex models that better mimic the host environment.

ROS are a group of chemically reactive oxygen-containing molecules or free radicals, including the superoxide anion (O_2_^−^), hydrogen peroxide (H_2_O_2_), and the hydroxyl radical (·OH), etc. ROS are ubiquitous in biological systems, being both by-products of normal metabolism and important signaling molecules (Kozlov et al., 2024). The accumulation of ROS leads to oxidative stress, causing an imbalance in the redox homeostasis within fungal cells, triggering oxidative damage, and affecting fungal development (Song, 2018; Song et al., 2018), even possibly achieving a sterilizing effect (Sui et al., 2021; Wong-Deyrup et al., 2021). Peroxisomes are key eukaryotic organelles involved in diverse metabolic processes, such as ROS detoxification and fatty acid β-oxidation (Kleiboeker and Lodhi, 2022; Kozlov et al., 2024; DiGiovanni et al., 2025). Peroxins are key components of peroxisomes and are responsible for regulating the biogenesis, maintenance, and function of peroxisomes (Sibirny, 2016; Akşit and van der Klei, 2018). Peroxins enable the selective import of peroxisome-targeted proteins into the lumen of the organelle (Cantelli et al., 2017; Akşit and van der Klei, 2018). In the present study, following SNH treatment of dermatophytes, peroxin gene (*PEX*) expression was significantly upregulated, which may be a response to the external stress caused by SNH. However, the expression of peroxins showed some differences between the two *Trichophyton* species. Furthermore, unpublished data from our research indicate that SNH elevates ROS levels in *C. neoformans*, consistent with the findings in mouse MH-S alveolar macrophages (Sacheli et al., 2021). On the other hand, *SOD* expression was markedly and continuously downregulated in response to SNH treatment (**Figures 4E**, **5E**), suggesting that SNH may inhibit SOD activity, thereby may impair intracellular ROS clearance. Further measurement of intracellular ROS levels under SNH treatment confirmed the alteration of redox balance (**Figure 6**). This disruption in redox balance likely leads to ROS accumulation and subsequent fungal damage, due to interaction of ROS with biological macromolecules (Song, 2018; Song et al., 2018). The precise mechanism underlying this effect remains unclear and warrants further investigation.

Carbon metabolism, which provides both energy and essential precursors for the synthesis of amino acids and nucleotides, occupies a central role in cellular metabolism (Liu et al., 2025). The transcriptomic analysis of SNH-treated dermatophytes at different time points revealed that DEGs were primarily enriched in pathways related to carbon and amino acid metabolism. Energy, as the core of life activities, profoundly influences diverse biological processes (Wijnants et al., 2022; DiGiovanni et al., 2025). Isocitrate lyase (ICL), a key enzyme of the glyoxylate cycle, participates in fatty acid and cholesterol metabolism in *T. rubrum*. This pathway bypasses several steps of the tricarboxylic acid (TCA) cycle, helping to sustain energy supply under carbon-limited conditions, those in the host stratum corneum, which is rich in lipids and keratin, thus facilitating colonization and proliferation (Cantelli et al., 2017; Kamal et al., 2021). Therefore, *ICL* has been recognized as a promising antifungal target (Kamal et al., 2021). Hexokinase (HK), catalyzing the first and rate-limiting step of glycolysis, critically influences glucose utilization and energy production. β-Glucosidase (GLU) hydrolyzes β-glycosidic bonds (such as, in cellulose derivatives) to generate glucose, directly contributing to carbon source breakdown and energy metabolism. During infection, β-glucosidase has been identified as a common processed antigen, potentially supporting early carbon metabolism by degrading glucose polymers present in the host stratum corneum (Eymann et al., 2018; Martins et al., 2020). Our results indicate that SNH treatment significantly reduced *HK* expression in the TM strain, suggesting that it impaired glucose utilization, causing inadequate energy supply, which may compromise detoxification capacity. Under these conditions, compensatory upregulation of *ICL* and *GLU* gene expression was observed, likely reflecting activation of alternative energy-generating pathways due to restricted glucose availability (**Figure 4**). In contrast, the clinical S136 strain exhibited sustained downregulation of *ICL* expression, accompanied by a compensatory increase in *HK* expression to maintain energy metabolism (**Figure 5**). We hypothesize that *ICL* may represent a potential target of SNH, though its role appears to exhibit species-specificity. The underlying mechanisms require further investigation.

We acknowledge several limitations of this study. First, the potential impact of strain-level heterogeneity was not examined. Whether the inhibitory effect of SNH is consistently observed across diverse strains remains to be determined. Therefore, future work should broaden the strain panel and evaluate clinical applicability, Therefore, future work should expand the strain panel and evaluate clinical applicability, particularly against resistant strains with diverse characteristics. Second, this study primarily focused on the *in vitro* inhibitory effects of SNH and evidence on *in vivo* efficacy is limited. Subsequent investigations should assess efficacy and safety to evaluate the therapeutic potential of SNH. Third, the precise molecular target(s) of SNH remain unidentified. Chemical biology, such as activity-based probes coupled with mass spectrometry, could directly identify interacting proteins and guide structure-based drug optimization. Fourth, combination therapy strategies and potential synergy with existing antifungal drugs have not been explored. Such studies may yield new regimens for treating refractory or recurrent dermatophytoses.

## 5 Conclusion

**Figure 7:**
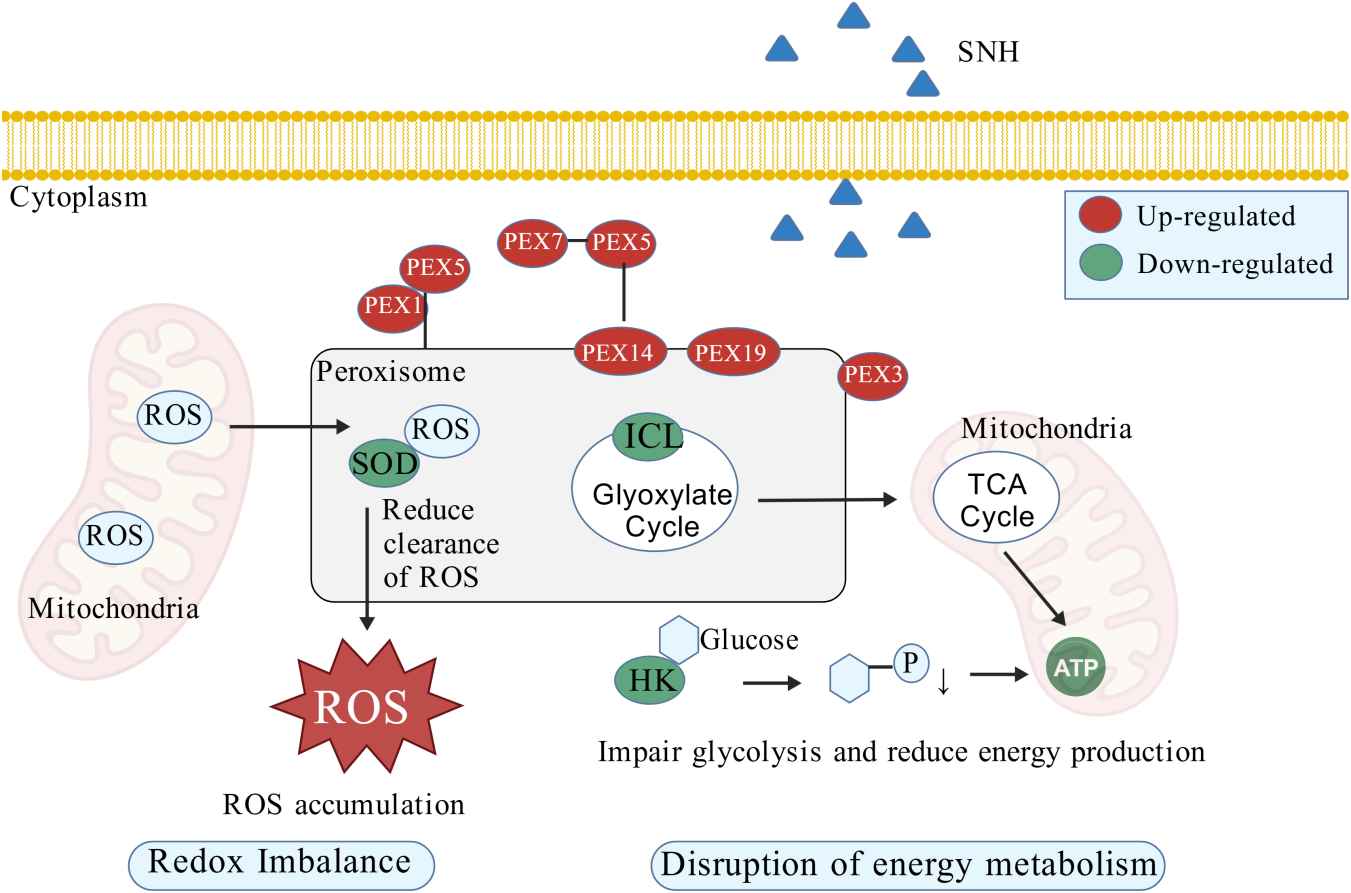
Mechanism of sodium new houttuyfonate- (SNH)-mediated antifungal activity against dermatophytes. SNH exerts its antifungal effects by disrupting redox homeostasis and energy metabolism in dermatophytes. In response to SNH stress, the expression of peroxisomal *PEX* genes, encoding peroxin proteins, is upregulated. Concurrently, SNH suppresses the expression of *SOD*, encoding superoxide dismutase, impairing the elimination of ROS generated during normal cellular activities, particularly under stress conditions. This leads to intracellular ROS accumulation, disruption of redox balance, and subsequent fungal oxidative stress damage. In addition, SNH inhibits the expression of genes encoding key energy metabolism enzymes, including *hexokinase* (*HK*) and isocitrate lyase (*ICL*). Reduced *HK* expression decreases glucose activation, thereby limiting glycolysis and ATP production. Downregulation of *ICL* restricts energy output from the glyoxylate cycle, a bypass route that feeds into the tricarboxylic acid (TCA) cycle while avoiding decarboxylation steps. The resulting energy deficiency, combined with ROS accumulation, collectively contributes to fungal oxidative stress damage. Created with BioGDP.com (Jiang et al., 2025).

In summary, this study demonstrates that SNH exhibits significant antifungal activity against dermatophytes, involving the inhibition of conidia germination rate, suppression of hyphal development, and reduction of fungal biomass production, collectively leading to marked growth restriction. At the molecular level, SNH disrupts cellular metabolism, particularly energy metabolism, and impairs fungal ROS-detoxification capacity. These effects ultimately result in disrupted redox homeostasis, accumulation of ROS, and oxidative stress damage, which collectively contribute to fungal growth inhibition (**Figure 7**). These findings provide important experimental evidence and a theoretical foundation for developing SNH as a promising candidate for anti-dermatophyte therapy.

## Data Availability

The datasets presented in this study can be found in online repositories. The names of the repository/repositories and accession number(s) can be found in the article/**Supplementary Material**.
