# Supplementary material for: Sodium new houttuyfonate inhibits the growth of Trichophyton spp. by potentially inducing oxidative stress and metabolic disruption
Source: Front Cell Infect Microbiol. 2026 Aug 5;16:1880130. doi: 10.3389/fcimb.2026.1880130 (PMC13485585; doi:10.3389/fcimb.2026.1880130)
Supplement: Supplementary file 1 [file Table1.docx]

Figure S1. Enrichment counts of differentially expressed genes in different pathways after 1 h of SNH treatment. (A) *Trichophyton mentagrophytes*; (B) *Trichophyton rubrum*.

Figure S2. Enrichment counts of differentially expressed genes in different pathways after 6 h of SNH treatment. (A) *Trichophyton mentagrophytes*; (B) *Trichophyton rubrum*.
