# Supplementary material for: Sodium new houttuyfonate inhibits the growth of Trichophyton spp. by potentially inducing oxidative stress and metabolic disruption
Source: Front Cell Infect Microbiol. 2026 Aug 5;16:1880130. doi: 10.3389/fcimb.2026.1880130 (PMC13485585; doi:10.3389/fcimb.2026.1880130)
Supplement: Supplementary file 2 [file Table2.docx]

Figure S3. (A) Quantitative analysis of ergosterol biosynthesis‑related genes in *Trichophyton mentagrophytes*. (B) Quantitative analysis of ergosterol biosynthesis‑related genes in *Trichophyton rubrum*. Data analysis was conducted using one-way analysis of variance (ANOVA). Significance levels: ns, *p* > 0.05; **p* < 0.05; ***p* < 0.01; ****p* < 0.001.
