# Supplementary material for: Sodium new houttuyfonate inhibits the growth of Trichophyton spp. by potentially inducing oxidative stress and metabolic disruption
Source: Front Cell Infect Microbiol. 2026 Aug 5;16:1880130. doi: 10.3389/fcimb.2026.1880130 (PMC13485585; doi:10.3389/fcimb.2026.1880130)
Supplement: Supplementary file 3 [file Table4.docx]

Table S2. Information on genes related to ergosterol synthesis.

| strain | gene | 0 h vs 1 h | | | 0 h vs 6 h | | | annotation |
| --- | --- | --- | --- | --- | --- | --- | --- | --- |
|  |  | FDR | log2FC | regulated | FDR | log2FC | regulated |  |
| TM | TMEN_3035 | 0.300 | -0.365 | normal | 0.168 | -0.440 | normal | Ergosterol biosynthesis ERG4/ERG24 family |
|  | TMEN_4188 | 0.406 | 0.204 | normal | 0.001 | 0.679 | normal | Ergosterol biosynthesis ERG4/ERG24 family |
|  | TMEN_987 | 0.666 | -0.109 | normal | 0.176 | -0.268 | normal | Erg28 like protein |
| TR | H107_01691 | 0.443 | 0.384 | normal | 0.271 | 0.341 | normal | short chain dehydrogenase(3-keto-steroid reductase erg27) |
|  | H107_01813 | 0.819 | 0.130 | normal | 0.206 | 0.412 | normal | Ergosterol biosynthesis ERG4/ERG24 family |
|  | H107_03526 | 0.131 | -0.688 | normal | 0.433 | 0.305 | normal | Erg28 like protein |
|  | H107_04739 | 0.508 | 0.275 | normal | 0.070 | 0.454 | normal | Ergosterol biosynthesis ERG4/ERG24 family |
|  | H107_05369 | 0.003 | -0.821 | normal | 0.058 | -0.398 | normal | Ergosterol biosynthesis ERG4/ERG24 family |
|  | H107_08034 | 0.864 | -0.081 | normal | 0.102 | 0.434 | normal | Ergosterol biosynthesis ERG4/ERG24 family |
|  | H107_08144 | 0.350 | -0.411 | normal | 0.173 | -0.464 | normal | Ergosterol biosynthesis ERG4/ERG24 family |

Note: TM, *Trichophyton mentagrophytes*; TR, *Trichophyton rubrum*
